# Artificial Intelligence Based Study Association between p53 Gene Polymorphism and Endometriosis: A Systematic Review and Meta-analysis

**DOI:** 10.1155/2022/8568820

**Published:** 2022-11-18

**Authors:** Xia Ma, Xiaoxiao Jin, Xiujuan Shao, Wanjing Hu, Haihong Jin, Yiqun Wang

**Affiliations:** ^1^Department of Obstetrics and Gynecology, Taizhou Hospital, Taizhou 317000, Zhejiang, China; ^2^Department of Obstetrics and Gynecology, Taizhou Women and Children's Hospital, Taizhou 318000, Zhejiang, China; ^3^Department of Obstetrics, Taizhou Hospital, Taizhou 317000, Zhejiang, China

## Abstract

**Background:**

The P53 gene is critical to the onset and progression of cancers. Currently, relevant study findings indicate that the p53 gene may have a strong association with the risk of endometriosis, but these findings have not been united. To gather more statistically meaningful clinical data, we used meta-analysis to examine the relationship between the rs1042522 single nucleotide polymorphism of the tumor suppressor gene p53 and the incidence of endometriosis.

**Methods:**

Through a comprehensive literature survey of PubMed, MEDLINE, EMBASE, Springer, and Web of Science literature databases, we obtained a clinical control case study on the relationship between p53 gene polymorphism and the prevalence of female endometriosis and finally traced the relevant references included. The quality of the literature included in this study was evaluated, and Revman5.3 was used to complete the meta-analysis.

**Results:**

This research includes eight publications. The total number of cases in the study group was 1551, whereas the total number of cases in the control group was 1440. The findings of the sensitivity analyses of each omitted piece of the literature revealed no significant difference. The results of the meta-analysis showed that there were significant differences in the GG gene frequency (OR = 0.56, 95%CI (0.38, 0.92), *P* = 0.003), allele G (OR = 2.46, 95%CI (1.41,4.29), *P* = 0.002), and allele C (OR = 0.62, 95%CI (0.46, 0.84), *P* = 0.002) between the study group and the control group (*P* < 0.01), but there was no significant difference in the GC gene frequency (OR = 1.17, 95%CI (1.01,1.36), *P* = 0.03), and the CC gene frequency (OR = 1.25, 95%CI (0.85,1.82), *P* = 0.26) (*P* > 0.01).

**Conclusion:**

Our study results show that there is a significant correlation between the single nucleotide of the p53 gene and the incidence rate of female endometriosis, in which the decrease of the GG gene frequency and the increase of allele C are likely to increase the risk of such diseases.

## 1. Introduction

**Figure 1:**
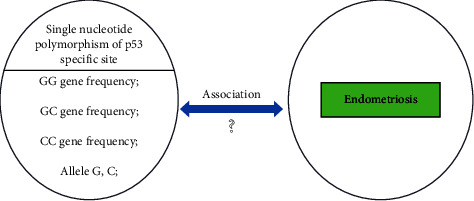
Possible association between a p53-specific single nucleotide polymorphism and endometriosis.

Endometriosis refers to a series of clinical symptoms caused by the presence of endometrial glands and stroma outside the uterine cavity. Although this kind of disease is usually benign, its incidence rate in the global female population is quite high, up to 10%–15%, and according to the relevant epidemiological survey results, this kind of disease is increasing year by year [1–3]. Although the clinical symptoms of endometriosis are benign, if we do not pay attention and get targeted treatment, it is likely to have invasion and pathological metastasis and eventually lead to more serious diseases [4–6]. Therefore, some clinicians and experts believe that endometriosis is a kind of tumor disease that is closely related to polygenic genetic factors [7–9]. Among them, the p53 gene is a kind of pathogenic gene that is strongly linked to tumor incidence, and its unique single nucleotide polymorphism is linked to the prevalence of female endometriosis. [10, 11] (see Figure 1). Therefore, many doctors and researchers have carried out clinical research on the problem and made research progress to varying degrees [12, 13]. However, there is no unified conclusion about the real intrinsic role of the p53 gene SNP and endometriosis. To better evaluate the relationship between the two, this study selected for meta-analysis the internationally published clinical control case studies on p53 gene polymorphism and female endometriosis prevalence from 2001 to 2022 and systematically evaluated the relationship between p53 gene polymorphism and endometriosis prevalence, in order to provide more evidence-based medical data for revealing the internal relationship between gene polymorphism and endometriosis prevalence. The report is as follows.

## 2. Materials and Methods

### 2.1. Data Sources and Literature Search Methods

PubMed, MEDLINE, EMBASE, Springer, and Web of Science literature databases were comprehensively searched through Computer artificial intelligence systems to obtain highly relevant literature related to this study. The language of literature retrieval is limited to English, and the retrieval period of literature is from 2001 to 2022. The strategies of literature retrieval are fast retrieval of English words and combinatorial retrieval of literature keywords. The key words were “endometriosis,” “rs1045552,” “p53 gene,” “SNP polymorphism,” “genetic variation analysis,” and “control test.” We can trace the complete text of the database by freely combining these keywords simultaneously, paired with manual retrieval to get further relevant reference material. The retrieval time is April 30, 2022.

### 2.2. Inclusion Criteria

① The included literature is the international published literature about the relationship between p53 gene polymorphism and female endometriosis incidence rate; ② the purpose and statistical methods of each study in the literature are highly similar; ③ the subjects were all patients with endometriosis confirmed by clinical diagnosis; ④ the genotype frequency distribution of patients in the control group conformed to Hardy–Weinberg law; ⑤ the study included the main outcome indicators set out in this paper, and the data used for analysis were complete.

### 2.3. Exclusion Criteria

① The literature does not provide specific research methods or complete data; ② the experimental group was nonendometriosis patients with other related diseases; ③ the genotype frequency distribution of patients in the control group did not meet Hardy–Weinberg law; ④ for the repeatedly published research content, only one piece of literature was introduced into this study.

### 2.4. Selection of Literature

Two studiers independently finished the screening of the literature. First, all literature titles and abstracts were independently read and analysed by these 2 studiers. The unfit for paper and report were then eliminated, and the fit for paper and report were collected and systematically reviewed by these two researchers. Following that, two researchers undertook cross-checking in order to exclude the questioned literature. Finally, a third studier was added to help in arbitration. In this literature, the NU1 questionnaire assessed general health, mental health symptomatology, use of alcohol, nicotine 23, cannabis, and other substances, including the nonmedical use of prescription substances; migraines and headaches; inattention 24; and baldness.

### 2.5. Data Extraction

Two studiers were assigned to independently and professionally extract the relevant data from this study. The data information mainly includes the following: the first author of the literature, the year of publication, the number of patients in the study group and the control group, and the age, gender, and physical condition of the patients included in this study. In this study, all data are independently analyzed and compared by two researchers. When there are significant disparities in the study data, a third-party research team will be assembled to undertake another round of systematic examination.

### 2.6. Literature Quality Assessment

For this study, the recommended criteria for evaluating genotype frequency and gene-disease association research were adopted [14]. First, two researchers were arranged to read and analyze independently according to the criteria for inclusion and exclusion of literature, and then representative literature was selected and timely literature was proposed with insufficient data sample size, poor quality, and high repetition. Finally, cross-check the literature; if there are differences again, arrange an on-site discussion or enlist a third party to determine whether to include them in this study. In this study, the Ottawa News Broadcasting Scale (NOS scoring method) was used to comprehensively evaluate the quality of each document. The higher the score of a document, the better the quality of the document and the more representative it is.

### 2.7. Statistical Analysis

Revman5.3 data meta-analysis software. First, the statistical heterogeneity of the literature included in this research was examined. When there is no statistical heterogeneity among the research findings (*P* > 0.1, *I*^2^ > 50%), the fixed effects model is used for analysis; when there is statistical heterogeneity among the research results (P 0.1, *I*^2^ > 50%), first examine whether the data included in the study is accurate. The full text of the literature is then carefully read to objectively evaluate and judge whether there is obvious clinical research heterogeneity or methodological heterogeneity in the literature; if there is a large heterogeneity, the random effects model is used to consolidate and analyze the data. The OR value and 95% CI were used as endometriosis incidence rate analysis markers in this research.

## 3. Results

### 3.1. Literature Search Method and Screening Process

**Figure 2:**
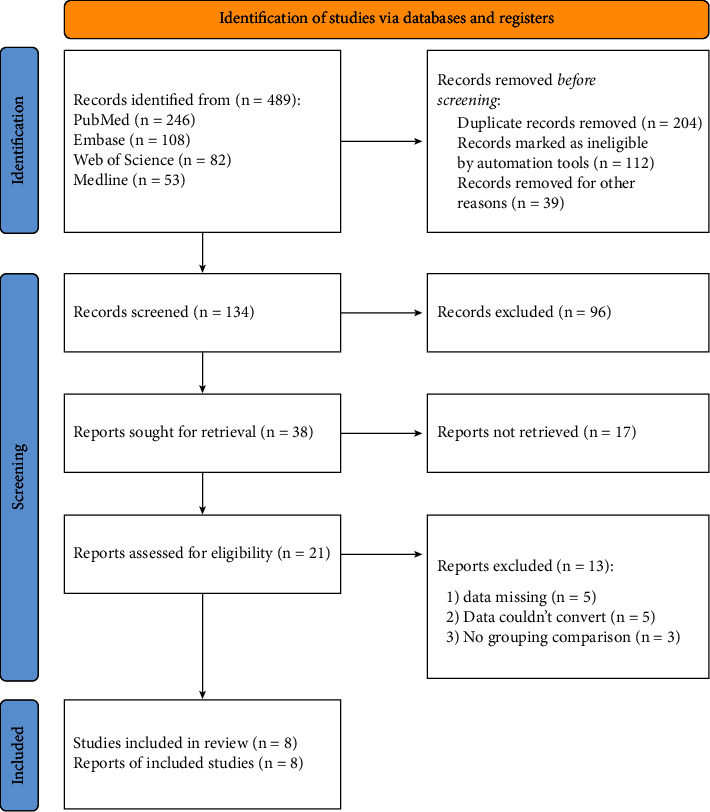
Flow chart of literature screening included in this study.

In this study, a total of 489 pieces of literature were obtained after preliminary screening. After reading the title, abstract, and full-text content of the literature, the literature that obviously did not meet the inclusion criteria, such as summary literature, case reports, and repetitive literature, was excluded. Finally, 8 pieces of literature with high quality and important representative significance were included, as shown in Figure 2. At the same time, the excluded literature and the main reasons for excluding this literature are listed in Table 1.

### 3.2. Literature Quality Evaluation

According to the retrieval scheme and document retrieval process described in 1.1 above, 8 articles were finally included in our study [17]. These articles included 2991 female patients with endometriosis, including 1551 patients in the experimental group and 1440 patients in the control group. There was no statistical significance in the age, weight, sex ratio, and family genetic history of female endometriosis patients. The NOS scoring standard was used to evaluate the treatment of the literature included in this study. This literature met the NOS scoring standard [12]. The evaluation results are shown in Table 2.

### 3.3. Meta-Analysis Results

#### 3.3.1. GG Genotype Frequency

**Figure 3:**
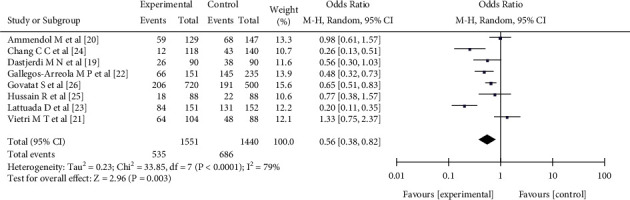
The correlation between the GG genotype frequency and female endometriosis incidence rate.

Eight pieces of literature [18–25] reported the relationship between the single nucleotide polymorphism GG genotype frequency at the rs1042522 site of the p53 gene and female endometriosis, including 1551 patients with female endometriosis and 1440 patients with nonendometriosis. There was heterogeneity in the literature (*I*^2^ = 79%, *P* <0.0001). The random effect model analysis showed that the GG genotype frequency of endometriosis patients in the experimental group was significantly lower than that in the control group, with a significant difference (OR = 0.56, 95%CI (0.38, 0.92), *P* = 0.003) (see Figure 3).

#### 3.3.2. GC Genotype Frequency

**Figure 4:**
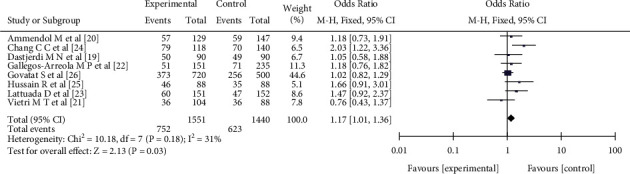
The correlation between the GC genotype frequency and female endometriosis incidence rate.

Eight pieces of literature [18–25] reported the relationship between the GC genotype frequency of a single nucleotide polymorphism at the rs1042522 site of the p53 gene and female endometriosis, including 1551 patients with female endometriosis and 1440 patients with nonendometriosis. There was no significant heterogeneity in the literature (*I*^2^ = 31%, *P* = 0.18). Fixed effect model analysis showed that there was no significant difference in the frequency of GC genotypes in the experimental group of endometriosis patients (OR = 1.17, 95%CI (1.01, 1.36), *P* = 0.03) (see Figure 4).

#### 3.3.3. CC Genotype Frequency

**Figure 5:**
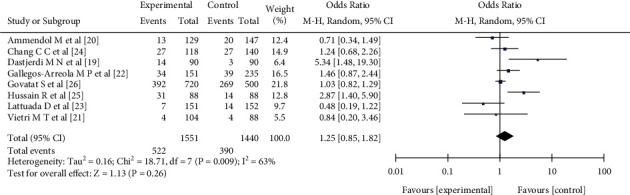
The correlation between the CC genotype frequency and female endometriosis incidence rate.

Eight pieces of literature [18–25] reported the relationship between the CC genotype frequency of the single nucleotide polymorphism at the rs1042522 of the p53 gene and female endometriosis, including 1551 patients with female endometriosis and 1440 patients with nonendometriosis. There was heterogeneity in the literature (*I*^2^ = 63%, *P* = 0.009). The random effect model analysis showed that there was no significant difference in the CC genotype frequency between the experimental group and the control group (OR = 1.25, 95%CI (0.85, 1.82), *P* = 0.26) (see Figure 5).

#### 3.3.4. Allele G

**Figure 6:**
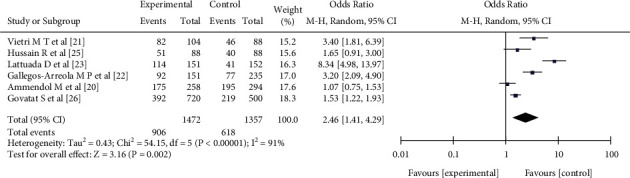
The correlation between allele G and female endometriosis incidence rate.

Six pieces of literature [19–25] reported the relationship between the single nucleotide polymorphic allele G at the rs1042522 locus of the p53 gene and female endometriosis, including 1472 patients with female endometriosis and 1357 patients with nonendometriosis. There was heterogeneity in the literature (*I*^2^ = 91%, *P* < 0.00001). The random effect model analysis showed that there was a significant difference in allele G between the experimental group and the control group, with a statistical significance (OR = 2.46, 95% CI (1.41,4.29), *P* = 0.002) (see Figure 6).

#### 3.3.5. Allele C

**Figure 7:**
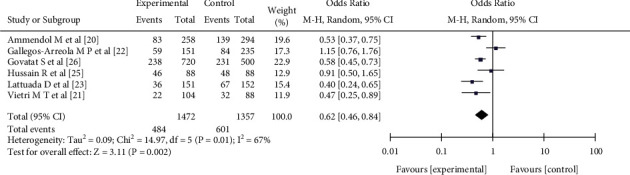
The correlation between allele C and female endometriosis incidence rate.

Six pieces of literature [19–25] reported the relationship between the single nucleotide polymorphism allele C at the rs1042522 locus of the p53 gene and female endometriosis, including 1472 patients with female endometriosis and 1357 patients with nonendometriosis. There was heterogeneity in the literature (*I*^2^ = 67%, *P* = 0.01). The random effect model analysis showed that there was a significant difference in allele C between the experimental group and the control group, with a statistical significance (OR = 0.62, 95%CI (0.46,0.84), *P* = 0.002) (see Figure 7).

#### 3.3.6. Analysis of Publication Bias

No publication bias analysis was performed because there were few articles in this study.

## 4. Discussion

Endometriosis is the most common and frequently occurring gynecological disease among women in the reproductive period. Clinically, such diseases mainly include dysmenorrhea, chronic intermittent pelvic pain, infertility, and other symptoms. At present, there is no completely effective treatment [26, 27]. Although most of these diseases have benign clinical manifestations, they are highly invasive and recurrent due to their wide range of incidence and diverse clinical pathological features [28, 29]. This has not only greatly affected the physical and mental health of women but also greatly reduced their quality of life. As a result, it is critical for these patients to uncover the molecular pathogenic process of this illness after doing more in-depth clinical research and thoroughly reviewing current research data. It may not only enhance the prognosis of female patients but also their overall quality of life. Endometriosis affects approximately 10% (190 million) of women and girls of reproductive age worldwide Early diagnosis and effective treatment of endometriosis are important, but in many cases (including in low- and middle-income countries), access to early diagnosis and effective treatment is difficult. More research and increased awareness are therefore needed worldwide to achieve effective prevention, early diagnosis, and better management of this disease.

In recent years, great progress has been made in the study of the pathogenesis of female endometriosis, but there is no unified conclusion on its essential pathogenesis. Many clinicians and experts generally believe that endometriosis is a kind of disease affected by a variety of environmental and genetic factors, such as tumor-related genes, environmental detoxification genes, immune-related genes, and hormone level-regulating genes, of which tumor-related genes are the most representative factors [30, 31]. Among tumor-related genes, the p53 gene, which functions as a tumor suppressor, is not only one of the most often altered genes in human malignant tumors but it is also linked to more than half of all human malignancies. Under normal physiological settings, this gene is not required for the human body. When human DNA is damaged or abnormally proliferated, the p53 gene is overexpressed. [32]. In recent years, many clinicians and scholars at home and abroad have carried out in-depth research on the relationship between the single nucleotide polymorphism of the rs1042522 and the incidence rate of endometriosis, and they have achieved significant research results [33, 34]. However, due to the difference in the genetic background among different races, their conclusions are inconsistent. For example, the research results of MP Gallegos Arreola et al. [21] suggest that the polymorphism of the rs1042522 single nucleotide at the special site of the p53 gene is closely related to the occurrence of endometriosis, while the research results of Shinya Omori et al. show that the polymorphism of rs1042522 single nucleotide at the special site of the p53 gene is not significantly related to the occurrence of endometriosis.

Endometriosis has significant social, public health, and economic implications. The intense pain, fatigue, depression, anxiety, and infertility associated with the disease reduce the quality of life. Endometriosis causes intolerable pain for some patients, preventing them from going to work or school. In this context, treating endometriosis could reduce school absences or improve the labour capacity of individuals. Dyspareunia due to endometriosis can result in the interruption or escape of sexual intercourse, thus affecting the sexual health of patients and/or their partners. Treatment of endometriosis will help patients enjoy their human rights to access the highest standards of sexual and reproductive hygiene, quality of life, and overall well-being, thereby empowering them. As a consequence, it is of tremendous value and therapeutic relevance to thoroughly examine all of the data using meta-analysis for these current study findings and the most recent studies.

In this study, after the selection and repeated demonstration of research topics and further condensing keywords, 8 highly representative pieces of research literature were effectively obtained. In the literature, various researchers have concentrated on reporting the relationship between the GG genotype frequency, the GC genotype frequency, the CC genotype frequency, allele G, and allele C in the single nucleotide polymorphisms at special sites of the p53 gene and endometriosis. Through further statistics and meta-analysis of these literature results, we found that there were significant differences between the study group and the control group in the GG gene frequency, allele G, and allele C of the special site rs1042522 of the tumor suppressor gene p53 (*P* < 0.01), but there were no significant differences in the GC gene frequency and the CC gene frequency between them (*P*> 0.01). As a consequence of our findings, there is a strong link between the single nucleotide polymorphism rs1042522 at the particular location of the p53 gene and endometriosis, with a drop in the GG gene frequency and an increase in allele C likely increasing the risk of this illness. On the other hand, for commonly used antibiotics, such as amoxicillin [35], ornidazole [36], etc., the efficacy of some natural drugs for this disease is also of concern [37].

## 5. Summary

In this systematic review and meta-analysis about the association between p53 gene polymorphism and endometriosis, a total of 8 pieces of literature were included. The results of our study show that the single nucleotide polymorphism of rs1042522 at the special site of the p53 gene is highly correlated with endometriosis. As a result, in future clinical research, doctors will be able to effectively combine these data to carry out more extensive and in-depth joint analysis, revealing the pathogenesis of such diseases and doing a good job at prevention in advance, eventually controlling the clinical malignant rate of endometriosis.

## Figures and Tables

**Table 1:** Excluded literature and the main reasons for exclusion (not all).

Serial number	Author	Date of publication	Reason for exclusion
1	Hsieh and Lin [15]	2006	Data cannot be effective transformed
2	Ying et al [16]	2011	Limited data

**Table 2:** Basic characteristics of included literature.

Serial number	Author	Study location	Date of publication	Total cases

1	Dastjerdi et al [18]	Isfahan, Iran	(2013)	180
2	Ammendol et al [19]	Rome, Italy	(2008)	376
3	Vietri et al [20]	Naples, Italy	(2007)	192
4	Gallegos-Arreola et al. [21]	Guadalajara, México	(2012)	386
5	Lattuada et al. [22]	Milano, Italy	(2004)	303

## Data Availability

The data used in this study are available from the author upon request.
